# The Breathing for Life Trial: a randomised controlled trial of fractional exhaled nitric oxide (FENO)-based management of asthma during pregnancy and its impact on perinatal outcomes and infant and childhood respiratory health

**DOI:** 10.1186/s12884-016-0890-3

**Published:** 2016-05-17

**Authors:** Vanessa E. Murphy, Megan E. Jensen, Joerg Mattes, Michael J. Hensley, Warwick B. Giles, Michael J. Peek, Andrew Bisits, Leonie K. Callaway, Kirsten McCaffery, Helen L. Barrett, Paul B. Colditz, Sean K. Seeho, John Attia, Andrew Searles, Christopher Doran, Heather Powell, Peter G. Gibson

**Affiliations:** Priority Research Centre GrowUpWell, University of Newcastle and Hunter Medical Research Institute, Level 2, West Wing, University Drive, Newcastle, NSW 2308 Australia; Paediatric Respiratory and Sleep Medicine Department, John Hunter Children’s Hospital, Newcastle, NSW Australia; Department of Respiratory and Sleep Medicine, John Hunter Hospital, Lookout Road, New Lambton Heights, Newcastle, NSW 2305 Australia; Department of Obstetrics and Gynaecology, Sydney Medical School Northern, University of Sydney, Royal North Shore Hospital, St Leonards, Sydney, NSW 2065 Australia; Sydney Medical School Nepean, University of Sydney, Nepean Hospital, PO Box 63, Penrith, NSW 2751 Australia; Birthing Unit, Royal Hospital for Women Randwick, Barker St, Randwick, NSW 2031 Australia; School of Medicine, University of Queensland, Brisbane, QLD Australia; Obstetric Medicine, Royal Brisbane and Women’s Hospital, UQ Health Sciences Building, Butterfield St, Herston, Brisbane, QLD 4029 Australia; Sydney School of Public Health, University of Sydney, Room 301F, Edward Ford Building A27, Sydney, NSW 2006 Australia; Perinatal Research Centre, UQCCR, University of Queensland, Butterfield St, Herston, Brisbane, QLD 4029 Australia; School of Medicine and Public Health, University of Newcastle, Newcastle, NSW Australia; Hunter Medical Research Institute, Kookaburra Circuit, New Lambton, Newcastle, NSW Australia; Priority Research Centre for Healthy Lungs, University of Newcastle and Hunter Medical Research Institute, Level 2, West Wing, University Drive, Newcastle, NSW 2308 Australia

**Keywords:** Asthma, Pregnancy, Perinatal, Exhaled nitric oxide, FENO, Asthma management, Exacerbation, Antenatal care

## Abstract

**Background:**

Asthma exacerbations are common during pregnancy and associated with an increased risk of adverse perinatal outcomes. Adjusting asthma treatment based on airway inflammation rather than symptoms reduces the exacerbation rate by 50 %. The Breathing for Life Trial (BLT) will test whether this approach also improves perinatal outcomes.

**Methods/design:**

BLT is a multicentre, parallel group, randomised controlled trial of asthma management guided by fractional exhaled nitric oxide (FENO, a marker of eosinophilic airway inflammation) compared to usual care, with prospective infant follow-up. Women with physician-diagnosed asthma, asthma symptoms and/or medication use in the previous 12 months, who are 12–22 weeks gestation, will be eligible for inclusion. Women randomised to the control group will have one clinical assessment of their asthma, including self-management education. Any treatment changes will be made by their general practitioner. Women randomised to the intervention group will have clinical assessments every 3–6 weeks during pregnancy, and asthma treatments will be adjusted every second visit based on an algorithm which uses FENO to adjust inhaled corticosteroid (ICS) dose (increase in dose when FENO >29 parts per billion (ppb), decrease in dose when FENO <19 ppb, and no change when FENO is between 19 and 29 ppb). A long acting beta agonist (LABA) will be added when symptoms remain uncontrolled. Both the control and intervention groups will report on exacerbations at a postpartum phone interview. The primary outcome is adverse perinatal outcome (a composite measure including preterm birth, intrauterine growth restriction, neonatal hospitalisation at birth or perinatal mortality), assessed from hospital records. Secondary outcomes will be each component of the primary outcome, maternal exacerbations requiring medical intervention during pregnancy (both smokers and non-smokers), and hospitalisation and emergency department presentation for wheeze, bronchiolitis or croup in the first 12 months of infancy. Outcome assessment and statistical analysis of the primary outcome will be blinded. To detect a reduction in adverse perinatal outcomes from 35 % to 26 %, 600 pregnant women with asthma per group are required.

**Discussion:**

This trial will provide evidence for the effectiveness of a FENO-based management strategy in improving perinatal outcomes in pregnant women with asthma. If successful, this would improve the management of pregnant women with asthma worldwide.

**Trial registration:**

Australian New Zealand Clinical Trials Registry ACTRN12613000202763.

## Background

Asthma is a common chronic disease among pregnant women, that affects more than 12 % of pregnant Australian women [1], and has an increasing prevalence worldwide [2]. Up to 45 % of women with asthma have exacerbations during pregnancy, requiring medical intervention [3]; with the 20–30 % of women with asthma who continue to smoke during pregnancy [3, 4], at an even greater risk of a severe exacerbation [3]. Asthma itself, as well as exacerbations, moderate to severe asthma [5, 6] and smoking [7] are significant risk factors for poor perinatal outcomes, including pre-eclampsia, preterm delivery, low birth weight, small for gestational age, neonatal hospitalisation and perinatal mortality [5, 8]. In addition, oral corticosteroid use, a common treatment for exacerbations, has been associated with preterm delivery [9, 10] and low birth weight [6]. Thus, an intervention which reduces the rate of exacerbations during pregnancy, may also improve perinatal outcomes, in both smokers and non-smokers. Notably, in a meta-analysis of prospective and retrospective cohort studies, the risk for preterm delivery, preterm labour and neonatal hospitalisation among women with asthma was increased in those without active asthma management, while there was no increased risk in women with active asthma management during pregnancy [5, 11]. This suggests that the effect of maternal asthma on some perinatal outcomes may be modifiable with “active” asthma management, and that asthma management during pregnancy, in both smokers and non-smokers, is important and necessary for the health of the mother and her child.

Regular monitoring of asthma during pregnancy is recommended [12] for two main reasons: (i) typically, one third of women experience an improvement, one third have no change, and one third have a subjective worsening of their asthma while pregnant [13]; and (ii) these changes are not easily predicted [14], which can complicate management. Current practice is to adjust asthma therapy following an assessment of symptoms and lung function; however, these measures are not always reflective of airway inflammation [15], which is the target of inhaled corticosteroid (ICS) therapy, suggesting that treatment decisions may be inappropriate when based on clinical assessment alone. Inflammation-guided therapy, known as ‘inflammometry’ [16], is a novel alternative. This strategy has resulted in a significant reduction in exacerbations in non-pregnant adults with asthma, where the adjustment of ICS doses was informed by a marker (or a surrogate marker [17]) of inflammation, such as sputum eosinophils [18] or fractional exhaled nitric oxide (FENO) [19]. Although FENO measurement is supported by the American Thoracic Society for clinical use to identify the need for ICS treatment, predict the response to ICS, and to adjust ICS doses in non-pregnant asthma [20], data is lacking to support its use in pregnancy, with only one ‘inflammometry’ trial in pregnancy published to date [21].

Our Managing Asthma in Pregnancy (MAP) Study was the first randomised controlled trial (RCT) to investigate the use of FENO-guided management during pregnancy and was conducted in 220 non-smoking women [21]. In the intervention group, ICS dose was adjusted monthly based on the result of the FENO test, which measures steroid sensitive inflammation, while symptoms and lung function (assessed by the Asthma Control Questionnaire, ACQ [22, 23]) were used to add long acting beta agonist (LABA) when uncontrolled symptoms remained. In the control group, monthly treatment changes were made based on symptoms and lung function (assessed by the ACQ) alone. There was a significant 50 % reduction in the primary outcome, exacerbations requiring medical intervention, in the FENO group, compared to the control group [21]. Moreover, this trial found that exposure to oral and inhaled corticosteroids was significantly reduced in the FENO intervention group [21]. While the trial was not powered for neonatal or infant outcomes, the results also indicated a reduction in neonatal intensive care unit (NICU) admissions (7.6 % vs 16.5 %) and a reduced odds of recurrent episodes of bronchiolitis (OR 0.08, 95 % CI [0.01, 0.62]) and recurrent croup (OR 0.12, 95 % CI [0.01, 0.99]) in the first 12 months of life (parent reported), in the intervention versus control group [24]. These promising results suggest that improving asthma management in pregnancy may have long term benefits to childhood health; however, these outcomes need to be further investigated in an adequately powered trial. The MAP study ‘control’ group also received substantially enhanced care, in comparison to the management typically provided by current practice, including monthly asthma assessments and telephone follow-ups with a trained asthma educator, provision of free asthma medications, and monthly dose adjustments; thus, the efficacy of FENO-based management is yet to be compared against usual asthma care during pregnancy.

The effects of FENO-based asthma management during pregnancy on perinatal and infant outcomes are yet to be investigated in an adequately powered trial. In addition, whether the intervention is effective in reducing exacerbations in pregnant women who smoke is unknown. Examining the cost-effectiveness and acceptability of the intervention in the clinical setting is warranted, in order to support the future implementation of FENO-based management into clinical practice.

Therefore, the primary aim of the Breathing for Life Trial (BLT) is to assess whether FENO-guided management of asthma during pregnancy reduces the rate of adverse perinatal outcomes among non-smokers and smokers, compared with usual best care. Secondary aims of the trial are to assess whether, compared to usual best care, FENO-guided management of asthma during pregnancy: (i) reduces exacerbations requiring medical intervention among women who smoke; (ii) reduces the rate of bronchiolitis, croup and wheeze leading to emergency department presentation or hospitalisation in the first year of infancy; (iv) is cost effective; and (iv) is acceptable to pregnant women, midwives and care-givers in the antenatal clinic setting.

## Methods/design

### Study design

**Fig. 1:**
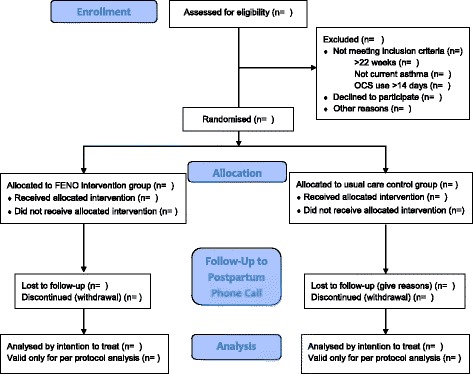
Participant flow diagram

**Fig. 2:**
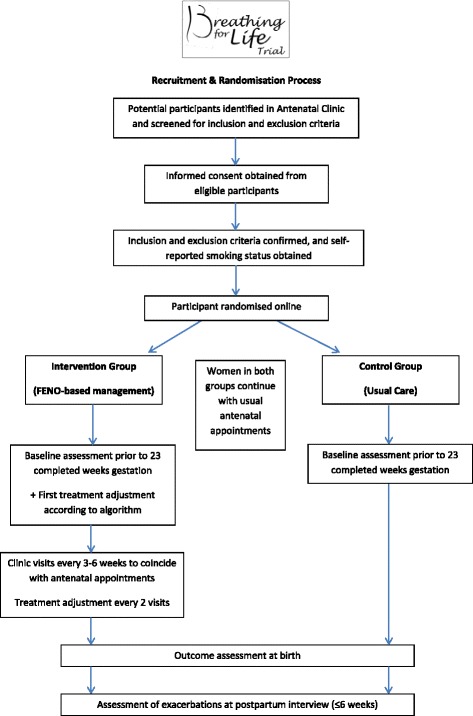
Breathing for Life Trial Study Design

BLT is a multicentre, parallel-group, RCT of FENO-guided management in pregnant women with asthma compared to usual care, with prospective infant follow-up for 12 months (Figs. 1 and 2). Women will be randomly allocated to one of two groups in a 1:1 ratio using blocks of four or six.

### Participants

#### Eligibility criteria

Pregnant women, aged 18 years or older, with physician diagnosed asthma, symptoms of asthma or use of asthma therapy (β_2_-agonist, ICS) in the past 12 months, and who are 12–22 weeks gestation (supported by ultrasound or clinical obstetric assessment) at randomisation will be enrolled in the trial. Exclusion criteria include the inability to attend monthly study visits, inability to perform manoeuvres required for spirometry or FENO, drug or alcohol dependence, chronic oral corticosteroid use for more than 14 consecutive days in the past three months, chronic lung disease other than asthma, or concomitant chronic illness which may affect participation (to be decided with medical investigator(s) on a case-by-case basis).

#### Setting

Eligible women with asthma will be recruited from public hospital antenatal clinics prior to 23 completed weeks gestation. This trial is being conducted within the antenatal clinic at six sites across Australia: the Royal Brisbane and Women’s Hospital (QLD), the Royal North Shore Hospital (Sydney, NSW), the Royal Hospital for Women Randwick (Sydney, NSW), Nepean Hospital (Penrith, NSW), The Canberra Hospital (Canberra, ACT) and the John Hunter Hospital (Newcastle, NSW, central site). If recruitment is slower than expected or difficulty is encountered with the aforementioned sites, other sites may be sought.

All women will provide written informed consent prior to participation.

### Intervention

#### Usual best care control group

Women randomised to the usual care (control) group will have one study visit of approximately 30 min at baseline with a trained research nurse or midwife. At this visit, patient demographics and characteristics (including maternal height, weight and ethnicity), asthma history and current medications will be collected. Asthma symptoms, triggers and self-management skills will be assessed; brief asthma education, correction of inhaler technique (as required), and written information (a pamphlet on asthma and pregnancy, available from Asthma Australia [25]) will be provided. Lung function will be assessed by spirometry, using the EasyOne Spirometer (Niche Medical, Sydney, Australia). Maternal smoking will be self-reported and confirmed by exhaled carbon monoxide (ECO) measurement (piCO Smokerlyzer Breath CO Monitor, Bedfont, UK; ≥10 parts per million (ppm) will be considered current smokers) and advice on smoking cessation provided as appropriate, with a referral to the Quitline for further assistance. Health care providers (general practitioner (GP), midwife and respiratory physician as applicable) will be informed of the woman’s participation in the trial. No medications will be provided by the trial for the control group; the woman’s asthma therapy will continue to be managed by their physician. All women will continue with standard antenatal care visits, and will receive a phone call within six weeks post-partum to enquire about any asthma-related health events (exacerbations) and adverse health events.

#### Treatment

Women randomised to the FENO-based management intervention will receive usual best care (as described above) plus monthly follow-up (three to six weekly) with the research nurse or midwife coinciding with their regular antenatal appointments, until delivery. Women without a written asthma action plan will be provided with one. At each visit, FENO will be measured using the portable NIOX MINO or NIOX VERO analyser (Aerocrine, Solna, Sweden), along with ECO measurement and spirometry. Asthma assessments will be conducted, including completion of the ACQ [22], a 7-item validated questionnaire which covers symptoms (6 items) and lung function (1 item), with scores >1.5 indicating uncontrolled asthma [23]. Results from the ACQ and FENO measurement will be entered into a predetermined algorithm (using an excel spreadsheet and/or specially-designed application for personal mobile devices), which will be used to assign any pharmaceutical treatment changes (Table 1). Starting at the first visit and every second visit thereafter, maintenance treatment changes will be made based on the results of this algorithm. When women are taking additional treatment for an exacerbation, no alterations will be made until they return to their maintenance dose. Women who self-report cigarette smoking in the previous week, or have ECO ≥10 ppm, will be considered current smokers for the purposes of applying the treatment algorithm. Required medication will be supplied by the hospital pharmacy at no cost to the participant. Asthma medications will be altered in a two-stage process: (i) assess FENO (eosinophilic inflammation) and adjust anti-inflammatory ICS; and (ii) assess symptoms and lung function (using the ACQ) and adjust long acting β_2_-agonists (LABA) (Table 1). When FENO is elevated (>29 ppb in non-smokers), the ICS dose will be increased by one dose step. When FENO is low (<19 ppb in non-smokers), the ICS dose will be decreased by one dose step. When FENO is in the mid-range (19–29 ppb for non-smokers), the ICS dose will not be changed. If the ACQ score is >1.5, that is, uncontrolled asthma, LABA will be added. Women using ICS or ICS/LABA combinations will have a treatment recommendation provided as an equivalent dose of budesonide (Pulmicort) or budesonide/e-formoterol (Symbicort). Budesonide has a category A safety rating for use in pregnancy [12, 26]. Women will use salbutamol (category A), as needed, for reliever medication. Exacerbation management will not be part of the trial. However, subjects will be provided with the phone contact details of the study nurse or midwife, and, if requesting advice during exacerbations, will be recommended to attend their primary care practitioner, or attend an emergency department.

**Table 1:** FENO Algorithm cut-points and dose changes based on FENO measurement on NIOX MINO and results from the Asthma Control Questionnaire (ACQ)

Non-smokers NIOX MINO cut-points	Smokers NIOX MINO cut-points	Symptoms [ACQ]	ICS dose change	β_2_-agonist dose change
>29 ppb	>22 ppb	N/A	↑ ICS x 1 step	No change
19–29	14–22	<=1.5	No change	No change
19–29	14–22	>1.5	No change	↑ LABA 1 step
<19	<14	<=1.5	↓ ICS x 1 step	No change
<19	<14	>1.5	↓ ICS x 1 step	↑ LABA 1 step

**Table 2:** Breathing for Life Trial Study Procedures

	Visit 1 (Control group)	Visit 1 (FENO group)	Follow-up Visits (FENO group)
Time frame	12–22^+6^ weeks gestation	12–22^+6^ weeks gestation	Every 3–6 weeks
Consent	✓	✓	
Inclusion/exclusion	✓	✓	
Other illnesses	✓	✓	
Asthma diagnosis	✓	✓	
Randomisation	✓	✓	
Demographics	✓	✓	
Height & weight	✓	✓	
Smoking questions	✓	✓	✓
Ethnicity	✓	✓	
Occupation	✓	✓	
FENO		✓	✓
Spirometry	✓	✓	✓
ECO	✓	✓	✓
ACQ		✓	✓
Asthma history	✓	✓	
Current medications	✓	✓	✓
Current symptoms	✓	✓	✓
Self-management education	✓	✓	✓
Written action plan		✓	
FENO treatment algorithm		✓	✓ (every 2nd visit)
Medication supply		✓	✓
Written information provided	✓	✓	
Letter to GP/primary carer	✓	✓	

### Additional methods

#### Infant follow-up

A validated parent-completed questionnaire [27] which examines patterns of respiratory illnesses and symptoms (such as croup, bronchiolitis, cough, colds and wheeze), family medical history, food intake, immunisations and household information will be mailed to consenting mothers from BLT with living children at 6 and 12 months of age. We have previously used this questionnaire to obtain data from the MAP study infants [24]. Medical records will also be used to assess emergency department presentations and hospitalisations for wheeze, bronchiolitis or croup.

#### Cost-effectiveness analysis

The interventions will be evaluated using the Assessing Cost-Effectiveness in Prevention (ACE-Prevention) methodology [28]. These methods are international best-practice for cost-effectiveness analysis and include: the adoption of a health system perspective; transparent and scientific methods to identify, measure and value both costs and outcomes from the trial; modelling and uncertainty testing of epidemiological and costing input parameters; and interpretation of results within a broader decision-making framework. Costing data will be collected using medical records and the measure of effect will be derived from the trial’s primary outcome.

#### Acceptance of the intervention

The acceptability of the FENO-guided management approach will be evaluated using questionnaires and qualitative interviews with pregnant women, midwives and antenatal clinic staff, to examine a range of clinical and patient perspectives. Face-to-face interviews at the home or in the antenatal clinic will be conducted with 15–20 pregnant women per group at 34–36 weeks gestation. Interviews will be analysed using qualitative descriptive methodology [29]. A purposive sample of women will be selected from different hospital sites, and from a range of backgrounds (smokers/non-smokers, severe/less severe asthma, high/low education). Interviews will also be conducted with the research nurse, a senior midwife and the nursing unit manager/other antenatal clinic staff to investigate specific issues related to a sustainable implementation of this intervention in each clinic (18–20 interviews total). Interviews will be digitally recorded and transcribed verbatim, and analysed thematically.

### Outcomes

#### Primary outcome

The primary outcome is the proportion of pregnancies with an adverse perinatal outcome, defined as: preterm birth (delivery at <37 weeks gestational age); intrauterine growth restriction (IUGR), defined as a birth weight <10th centile for gestational age (based on a dataset of 12,500 Australian births, calculated using the Gestation network calculators available online at http://gestation.net); perinatal mortality (stillbirth or neonatal death within the first 28 days of life); or neonatal hospitalisation at birth. These details will be determined by review of the hospital medical records and/or data extraction from the hospital obstetric/perinatal database (outcome assessor will be blinded to group allocation).

#### Secondary outcomes

Secondary outcomes for the trial are: (i) preterm birth, as previously defined; (ii) IUGR, as previously defined; (iii) perinatal mortality, as previously defined; (iv) neonatal hospitalisation, including number of days of admission; (v) mean birth weight; (vi) maternal hospitalisation for asthma, including number of days of admission; and (vii) in women who smoke, maternal exacerbations requiring medical intervention, defined as hospitalisation, emergency department presentation or a course of oral corticosteroids (severe exacerbation) or an unscheduled doctor’s visit (moderate exacerbations). These outcomes will be determined by review of the woman’s hospital medical records and/or data extraction from the hospital obstetric/perinatal database, and participant report at a postpartum telephone interview. The type of preterm birth will also be recorded (spontaneous labour, preterm rupture of membranes, or indicated preterm birth) [30].

### Additional outcomes

#### Infant follow-up

The outcome will be the proportion of subjects with severe respiratory illnesses (bronchiolitis, croup, wheezing) in the first year of life, defined as those that required an emergency department presentation or hospitalisation. This information will be determined from the parental questionnaires at 6 and 12 months and from medical records at each site.

#### Cost-effectiveness

The cost-effectiveness analysis will adopt adverse perinatal events as the primary outcome, consistent with the clinical trial.

#### Acceptance of the intervention

A qualitative description of the acceptability of the trial by a sub-sample of participants and participating antenatal clinic and staff will be collected.

### Sample size

#### Primary outcome

Previous data from pregnant smokers (*n* = 47) and non-smokers (*n* = 211) with asthma, demonstrated that 35.3 % of pregnancies managed by a clinical guidelines-based algorithm had an adverse perinatal outcome (preterm delivery, IUGR [birth weight < 10th centile], stillbirth, or neonatal intensive care [NICU] admission) compared to 26.2 % of those managed by a FENO-based algorithm (unpublished data). To demonstrate this group difference in the proportion of adverse perinatal outcomes (preterm birth, IUGR, neonatal hospitalisation, or perinatal mortality), with 90 % power and alpha of 0.05, between FENO-based management and usual best care, we require 539 women per group. Allowing for 10 % loss to follow-up, we aim to recruit 600 women per group.

#### Secondary outcome

We anticipate that a sample size of 600 women per group will include approximately 120 current smokers per group (20 %). This will provide 99 % power to detect a 50 % reduction in the proportion of women with exacerbations requiring medical intervention among smokers (as demonstrated in non-smokers in our previous RCT [21]). This represents a reduction from 52 % of women having exacerbations [3] to 26 %.

### Randomisation/treatment allocation

#### Sequence generation

The randomisation sequence will be computer generated using an online system accessed via a secure website, developed specifically for BLT by Hunter Medical Research Institute (Newcastle, NSW). Eligible consenting women will be randomly allocated to the FENO-based management or usual care groups using variable block sizes of 4 and 6, stratified by site and baseline self-reported smoking status.

#### Allocation concealment

The online system will not allow randomisation of a participant unless all criteria are met and will not allow unregistered withdrawal after randomisation. This system assigns each participant a unique randomisation number.

#### Implementation

The research nurse/midwife at the site will enrol the participants and enter their inclusion criteria, site and smoking status into the online system.

### Blinding

Given the nature of the intervention, it is not possible to blind participating women, the research staff or the participants’ healthcare providers, to their group allocation. However, the primary and secondary outcomes will be assessed by a review of medical records and data extracted from hospital databases by a researcher blinded to group allocation; there will be no involvement of research staff in the clinical management of pregnancy, labour and delivery; and analysis of the primary outcome will be performed by a statistician blinded to group allocation.

### Statistical methods

Data will be analysed on an intention-to-treat basis, using two-sided tests with *p* < 0.05 considered statistically significant.

#### Primary outcome

The proportion of subjects with an adverse perinatal outcome will be compared between groups using logistic regression. The outcome in the model will be adverse perinatal outcome (yes/no), the main predictor of interest will be management group and study centre will be included as a covariate, due to potential differences in socioeconomic status, environmental exposures and management practices.

#### Secondary outcomes

Mean birth weight will be compared between groups by linear regression, while the proportion of subjects with preterm birth, IUGR, perinatal mortality or neonatal hospitalisation will be compared using logistic regression, adjusted for study centre. The proportion of women requiring a hospitalisation for asthma and the proportion of smokers with an asthma exacerbation requiring medical intervention, will be compared between groups using logistic regression, adjusted for site.

### Additional outcomes

#### Infant follow-up

The proportion of infants with severe respiratory illnesses requiring an emergency department visit or hospitalisation in the first year of life will be compared between the groups using logistic regression, adjusted for site, and other potential confounders.

#### Cost-effectiveness

The analysis will model costs and outcomes over a ten year period discounting future costs and health outcomes at a rate of 3 % per year. The costs and health outcomes are summed to determine the incremental cost-effectiveness ratio. Monte Carlo analysis is used to derive 95 % uncertainty intervals. The results of the cost-effectiveness analysis will be considered in the context of other decision making criteria including: strength of evidence, capacity of the intervention to reduce inequity, acceptability to stakeholders, feasibility, sustainability and potential for other consequences. A sensitivity analysis will be conducted on parameters with uncertain values.

#### Acceptance of the intervention

Thematic analysis will be used to examine acceptability and barriers to implementation in the antenatal clinic.

### Data monitoring and safety board

An independent Data Monitoring and Safety Board (DSMB) will be established with terms of reference.

## Discussion

Women with asthma are at an increased risk of adverse perinatal outcomes. We propose that providing improved antenatal care, which incorporates optimum asthma management using a FENO-based approach, will be effective in reducing these risks among this population of women, with positive consequences for infant health both in the short term and long term. This trial will provide the first evidence as to whether the provision of FENO-based management, as opposed to current best care, can significantly reduce the rate of adverse perinatal outcomes, that is, preterm delivery, IUGR, perinatal mortality or neonatal hospitalisation. If proven successful, this intervention would have profound implications for the health of pregnant women with asthma and their offspring. Specifically, if severe respiratory illnesses such as bronchiolitis were reduced in infancy, then FENO-based asthma management in pregnancy has potential as a primary prevention strategy for childhood asthma, representing a significant breakthrough in asthma research. Furthermore, if successful and cost-effective, this intervention could be implemented into primary care settings such as the antenatal clinic and potentially reduce the burden on the health care system.

The trial will be powered to detect a significant difference in the proportion of smoking mothers who have exacerbations requiring medical intervention. This is an important objective for the following reasons: (i) pregnant women with asthma are more likely to smoke than pregnant women without asthma [4]; (ii) compared with non-smokers, asthmatic women who smoke are at an increased risk of poor perinatal outcomes from the combined effects of smoking, asthma and severe asthma exacerbations [3]; and (iii) the quit rate for smoking in pregnancy is surprisingly low (6–10 %) in Australia, despite best practice [31]. Therefore, an intervention that could reduce the rate of asthma exacerbations in women who smoke during pregnancy would have a significant impact on the management of a group of high-risk women.

The Breathing for Life Trial builds upon our previous trial, which successfully demonstrated a significant reduction in maternal exacerbations using FENO-based management. BLT simplifies the FENO-based management approach used in the previous trial in several ways: (i) the number of drug formulations available will be reduced; (ii) the FENO algorithm results will be generated electronically; (iii) asthma assessments will be aligned with antenatal clinic appointments; and (iv) fewer treatment changes will be made (every two months instead of monthly). In addition, we will measure FENO using the NIOX MINO (or NIOX VERO) electrochemical analyser, an instrument which is practical for use in the antenatal clinic setting, where space and time are limited. It is small and portable, relatively inexpensive and easy to use (no calibration required) and obtains a measurement in less than two minutes (Table 2).

The implementation of the approach in a public hospital antenatal clinic was not previously investigated, nor was the cost-effectiveness of the intervention. In this trial, we have chosen centres across NSW, ACT and Queensland which differ in location (capital city, regional, tertiary/non-tertiary hospital), the proportion of smoking and obese women, maternal age and education attainment, severity of asthma, ethnic backgrounds and socioeconomic status. Therefore, it is anticipated that the intervention will be applicable to the general population.

As part of this trial, we have included a cost-effectiveness analysis of the primary outcome, that is, adverse perinatal outcomes. This will provide policy relevant information that can be used to determine the resources required for wider implementation of the intervention. In addition, a qualitative assessment of the acceptability of the intervention to both mothers and the antenatal clinic staff will be performed. Therefore, this novel trial will address potential obstacles to implementation in standard practice, including issues of cost and feasibility, generalisability, and effectiveness, compared to usual care.

We acknowledge that our control group may not be reflective of typical usual care; however, it was deemed unethical to deny a certain level of care to women with asthma during pregnancy. Therefore, the control group will be provided with minimal assessment and education with a trained nurse/asthma educator at the first visit only, that is, self-management skills, knowledge and medication use will be assessed, with brief asthma education, correction of inhaler technique (as required), and written information provided. Of note, this is a reduction in the level of care given to the ‘control’ group compared to the MAP trial; thus, this minimal level of care is unlikely to affect group differences in our chosen outcomes.

In conclusion, the Breathing for Life Trial is designed to test an approach which is simple, effective in improving maternal asthma during pregnancy (both for smokers and non-smokers), whilst also being beneficial to the infant, cost-effective, accessible and acceptable to women attending antenatal clinics.

### Ethics approval and consent to participate

Ethics approval was obtained for the Breathing for Life Trial at all centres through the Hunter New England Human Research Ethics Committee (Reference Number 12/10/17/3.04, NSW HREC Reference No: HREC/12/HNE/357). Site-specific approval will be attained for all participating sites prior to active recruitment. Site-specific approvals have been obtained for the co-ordinating site at the John Hunter Hospital (SSA Reference No: SSA/12/HNE/393), and five external sites: Royal Brisbane and Women’s Hospital (SSA/14/QRBW/358), the Royal Hospital for Women Randwick (SSA 14/G/333), Nepean Hospital (SSA Reference No: SSA/14/NEPEAN/84), Royal North Shore Hospital (SSA/15/HAWKE/142) and The Canberra Hospital (ACT Health Human Research Ethics Committee, ETH.11.15.232). Ethics approvals have also been registered with the University of Newcastle (Reference No: H-2012-0422) and University of Queensland (Approval No: 2014000807) Human Ethics Committees. All participants will provide written informed consent prior to participation.

### Consent for publication

Not applicable

### Availability of data and material

All data supporting the findings will be deposited in the University of Newcastle’s institutional digital repository.

## Abbreviations

CI: confidence interval
FENO: fractional exhaled nitric oxide
GP: general practitioner
ICS: inhaled corticosteroid
LABA: long-acting beta-agonist
OR: odds ratio
RCT: randomised controlled trial
RR: relative risk
